# Performance simulation of the perovskite solar cells with Ti_3_C_2_ MXene in the SnO_2_ electron transport layer

**DOI:** 10.1038/s41598-024-56461-z

**Published:** 2024-03-08

**Authors:** Mahdiyeh Meskini, Saeid Asgharizadeh

**Affiliations:** Faculty of Physics, University of Tabriz, Tabriz, Iran

**Keywords:** Perovskite solar cell, Ti_3_C_2_ MXene, SCAPS-1D, Electron transport layer, Photocurrent, Built-in potential, Ideality factor, Optical physics, Optics and photonics

## Abstract

MXenes, a class of two-dimensional (2D) transition metal carbides and nitrides, have a wide range of potential applications due to their unique electronic, optical, plasmonic, and other properties. SnO_2_–Ti_3_C_2_ MXene with different contents of Ti_3_C_2_ (0.5, 1.0, 2.0, 2.5 wt‰), experimentally, has been used as electron transport layers (ETLs) in Perovskite Solar Cells (PSCs). The SCAPS-1D simulation software could simulate a perovskite solar cell comprised of CH_3_NH_3_PbI_3_ absorber and SnO_2_ (or SnO_2_–Ti_3_C_2_) ETL. The simulation results like Power Conversion Efficiency (PCE), Open circuit voltage (V_OC_), Short circuit current density (J_SC_), Fill Factor (FF), and External Quantum Efficiency (EQE) have been compared within samples with different weight percentages of Ti_3_C_2_ MXene incorporated in ETL. Reportedly, the ETL of SnO_2_ with Ti_3_C_2_ (1.0 wt‰) effectively increases PCE from 17.32 to 18.32%. We simulate the role of MXene in changing the ideality factor (n_id_), photocurrent (J_Ph_), built-in potential (V_bi_), and recombination resistance (R_rec_). The study of interface recombination currents and electric field shows that cells with 1.0 wt‰ of MXene in SnO_2_ ETL have higher values of ideality factor, built-in potential, and recombination resistance. The correlation between these values and cell performance allows one to conclude the best cell performance for the sample with 1.0 wt‰ of MXene in SnO_2_ ETL. With an optimization procedure for this cell, an efficiency of 27.81% is reachable.

## Introduction

Methylammonium lead iodide (CH_3_NH_3_PbI_3_) was used in solar cells for the first time in 2009 by Miyaska et al. ^1^. Metal halide perovskites have unique properties that justify their use in solar cells ^2,3^. Technological progress in the field of organic–inorganic solar cells since the last decade has revolutionized the research field to achieve a better alternative to conventional energy sources. That is why the circle of research has been expanded mainly to these energy sources due to the better results. Many efforts have already been made leading to achieving higher power conversion efficiency (PCE) with much cheaper fabrication cost ^4^. Perovskite solar cells (PSCs) have risen to stardom owing to their peculiar characteristics such as high charge carrier mobility, long free carrier diffusion length, broad and strong optical absorption, low exciton binding energy, as well as their cost-effective and easy solution process manufacture ^5,6^, Hybrid perovskites have great potential as being efficient, low-cost, and flexible materials for photovoltaic technology. Recent advancements have resulted in improved device stability and overall efficiency^7^. Metal halide perovskites are denoted by ABX_3_, where A refers to an organic cation, B to a metal cation, and X to a halogen anion ^8^. PSC consists of an electron transport layer (ETL) as an electron collector and a hole transport layer (HTL) that effectively extracts holes from the perovskite absorber layer ^9^. In PSCs, cell performance can be optimized by finding the best combination of ETL and HTL ^4^. The effect of suitable HTLs is also significant as they influence the extraction and contribution to the instantaneous flow of light-generated holes from the perovskite absorber layer to the PSC cathode. The use of highly pure Spiro-OMeTAD HTL has been widely accepted in manufacturing and stability factors. At the same time, the selection of ETL is also necessary to reduce the recombination rate as well as to optimize the efficiency of PSC ^10^. There must be a much better alignment in the energy bands between the HTL and the absorber layer to allow the transport of holes from the perovskite absorber layer to the HTL. The built-in (V_bi_) electric field created due to the choice of contact metals in the back and front contacts helps to maintain an electric field throughout the device configuration, which enables smooth transport of charge carriers throughout the device ^9^. Researchers have been working to improve PSC performance for decades. However, the proper selection of materials related to the optimal thickness in the device structure is a much-needed approach to increase device efficiency ^11^. SnO_2_ has emerged as a promising ETL in PSCs due to its optical transparency and environmental stability ^12–15^. In addition, the combination of SnO_2_ with n-type semiconductors or highly conductive materials is an effective method for further enhancing the electrical conductivity of the ETLs ^16–18^. This improvement has led to an increase in the PCE of the solar cells ^19–21^. Recently, two-dimensional (2D) materials have been gaining significant attention as potential candidates for use in photovoltaic PSCs due to their unique optical and electronic properties ^22–24^. A new family of 2D materials, known as MXenes, has emerged. These materials are composed of transition metal carbides, nitrides, and carbonitrides, with a general formula of M_n+1_X_n_T_x_ (where n can be 1, 2, or 3). In this formula, M represents a transition metal like titanium or vanadium, while X stands for carbon or nitrogen. T_x_ refers to the surface-terminating functional groups. These materials have found significant applications in PSCs ^25–27^. The first studies on MXene’s applications in PSCs date back to 2018, when they were used in absorber layers and ETLs ^28,29^. MXenes have been applied in the structure of PSCs to enhance their (PCE) ^30^. Surface termination in these materials can affect the density of states (DOS) and work function (WF) ^31,32^, offering new opportunities for PSC applications. Among the MXenes, Ti_3_C_2_ is an excellent additive for the SnO_2_ ETL, which is commonly used in PSCs ^33^. Films of SnO_2_ with different Ti_3_C_2_ contents (0.0, 0.5, 1.0, 2.0, 2.5 wt‰) were prepared by spin-coating onto indium tin oxide (ITO) substrates ^34^. Photovoltaic devices were constructed using an architecture consisting of ITO/ETL/CH_3_NH_3_PbI_3_/Spiro-OMeTAD/Ag.

It has been reported that incorporating MXenes into the ETL can decrease interface recombination, leading to higher PCEs for PSCs ^35^. It is useful to estimate the dominant recombination pathway to demonstrate the role of MXenes in influencing interfacial recombination. The ideality factor (n_id_) is a parameter used to determine the dominant recombination mechanism in a semiconductor device ^28^. One common method to calculate the nid is by measuring the open-circuit voltage (V_OC_) of the light intensity ^36^. A n_id_ value of 1 indicates that the dominant recombination mechanism at play is the interface Shockley–Read–Hall (SRH) recombination ^37^. On the other hand, a n_id_ value close to 2 suggests that the absorber layer’s dominant recombination mechanism is trap-assisted recombination ^38,39^. The n_id_s between unity and two will predict interfacial and bulk recombination superposition.

Impedance spectroscopy (IS) is a versatile technique used to monitor electrical and electrochemical processes and profile the electronic structure in devices. During an IS measurement, a small-signal, sinusoidal electrical stimulus is applied to a sample, and its response is monitored at different frequencies ^40^. This technique is used to measure the resistive and capacitive behavior of an electrochemical system. This is done by applying an alternating current (AC) potential to the system at different frequencies and then measuring the alternating current response through the cell ^41^. IS technique includes plotting the so-called Nyquist graph that illustrates the imaginary part of the complex impedance versus the real part of it. Fitting this graph, one could use the charge recombination resistance (R_rec_) in the equivalent circuit ^42^. In this study, we used IS to examine charge dynamics in the absorber layer and interfaces in simulated solar cells. This study aimed to investigate the changes in the IS response of PSCs and to identify the mechanisms responsible for the decrease in cell efficiency. Also, we calculated series resistance (R_S_) in the equivalent circuit ^43^. We used simulation software called SCAPS-1D ^44^ to investigate how adding different weight percentages of Ti_3_C_2_ MXene to the SnO_2_ ETL layer in a PSC structure (ITO/SnO_2_ (ETL)/CH_3_NH_3_PbI_3_/Spiro-OMeTAD/Ag) affected the photocurrent density (J_Ph_), n_id_, and R_rec_. The software considers the production and recombination of charge carriers in the layers and interfaces. Additionally, we studied the performance of PSCs with 1.0 wt.‰ MXene-assisted ETL for various thicknesses of the absorber and charge transport layers to optimize the structure and achieve the highest possible PCE.

## Methodology and simulations

SCAPS-1D is a software used for one-dimensional simulations. It calculates energy bands, current–voltage characteristics, and external quantum efficiency by solving continuity equations for electrons and holes and the Poisson equation ^45^. The software can also calculate recombination profiles and electric field distribution for layers and interlayers. The basic continuity equations used by this software for electrons and holes are:

1 $${J}_{n }= \text{ } qn{\mu }_{n}E + \text{ } q{D}_{n}\frac{\partial n}{\partial x} ,$$

2 $${J}_{p }= \text{ } qp{\mu }_{p}E-q{D}_{p}\frac{\partial p}{\partial x} ,$$

where ${\mu }_{n}$ and ${\mu }_{p}$ are the electron and hole mobility respectively, ${D}_{n} ({D}_{n})$ is the electron (hole) diffusion coefficient, $E$ is the electric field, $q$ is the electron charge, and $p (n)$ is the hole (electron) density. The recombination rate of electrons and holes $({U}_{n, p })$ can be calculated through the equations mentioned above:

3 $$\frac{\partial {J}_{n } \, }{\partial x} +G-{U}_{n}\text{ (}n\text{, }p\text{) = 0,}$$

4 $$-\frac{\partial {J}_{p } \, }{\partial x} +G-{U}_{p}\text{ (}n\text{, }p\text{) = 0,}$$

where $G$ is the electron–hole generation rate.

**Figure 1:**
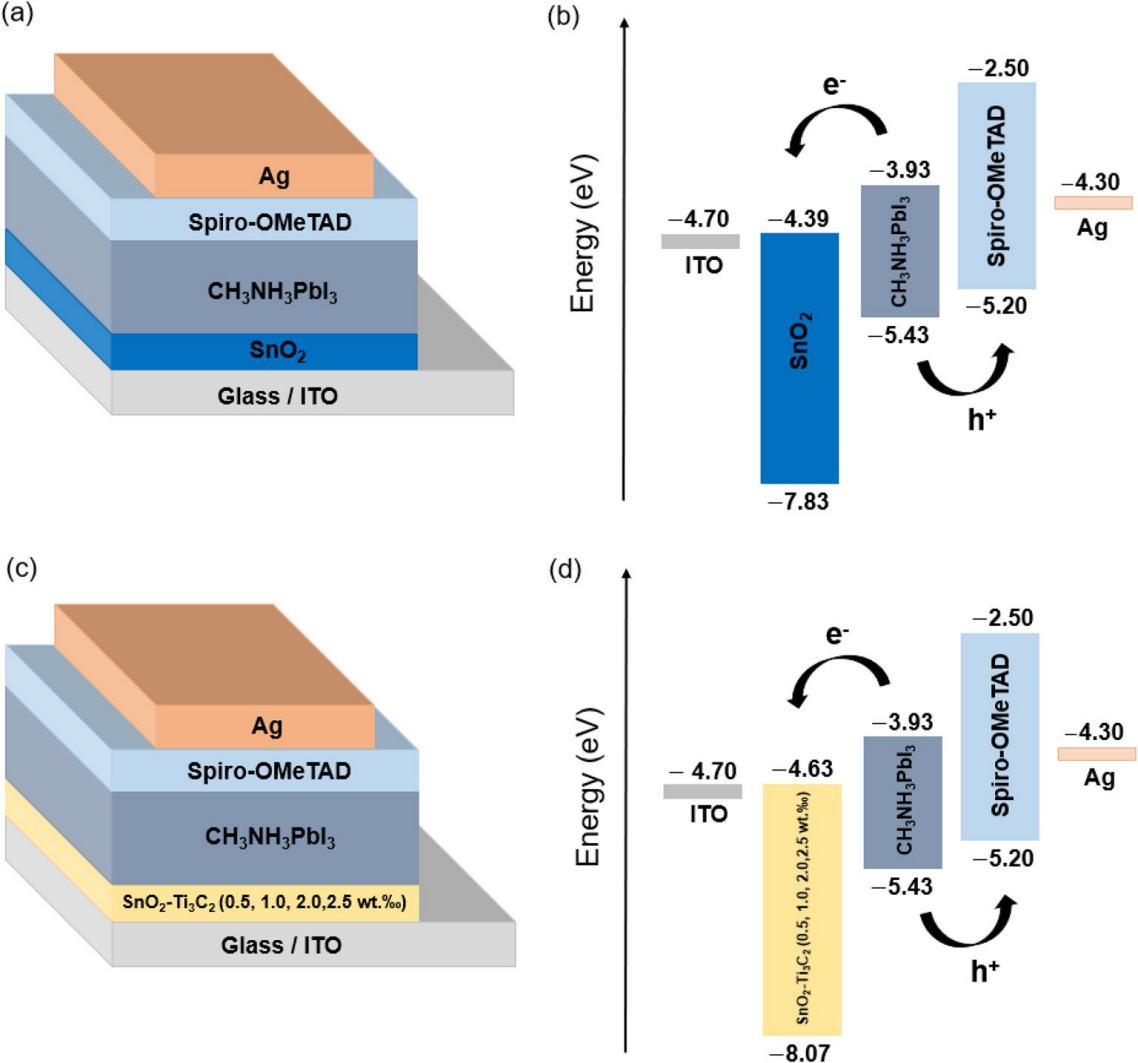
(**a**) Device architecture of ITO/SnO_2_/CH_3_NH_3_PbI_3_/Spiro-OMeTAD/Ag, and (**b**) its schematic energy level diagram. (**c**) Device architecture of ITO/SnO_2_-Ti_3_C_2_/CH_3_NH_3_PbI_3_/Spiro-OMeTAD/Ag, and (**d**) its schematic energy level diagram.

In a recent experiment, researchers incorporated Ti_3_C_2_ MXene in SnO_2_ ETL to enhance the efficiency of PSCs. To understand how this enhancement is achieved, simulations were conducted on two types of PSCs. The first one had the architecture of ITO/SnO_2_/CH_3_NH_3_PbI_3_/Spiro-OMeTAD/Ag, while the second one had the architecture of ITO/SnO_2_–Ti_3_C_2_ (0.5, 1.0, 2.0 and 2.5 wt.‰)/CH_3_NH_3_PbI_3_/Spiro-OMeTAD/Ag (Fig. 1). The simulation work used data provided by the original experimental work ^44^.

The material parameters for the pristine sample are selected from published experimental data and listed in Table 1. Interfacial parameters for simulation are shown in Table 2. In this table, N_A_ and N_D_ denote acceptor and donor densities, ε is relative permittivity, χ is electron affinity, E_g_ is band gap energy, and N_t_ is defect density. N_C_ and N_V_ are the effective densities of conduction and valance band states, respectively. To estimate the thickness of the layers, the SEM image provided in the experimental work ^34^ was used. In addition, the electron/hole thermal velocity for each layer was set to 10^7^ cm/s, simulated light conditions were AM1.5G, and the simulation temperature was 300 K. According to a study ^28^, incorporating Ti_3_C_2_ MXene into the layers of PSC does not affect the band gap energy. However, it does reduce the WF in the layers, which changes the electron affinity of the layers ^28^. This means that the band gap energy will remain the same for MXene-added structures. Moreover, the electron affinity of the MXene-added ETL has been measured to be 4.63 eV ^34^. It has been reported that the efficiency enhancement of PSCs is mainly due to the increased mobility of the layers when MXene is added. In this work, we have adopted an electron mobility of 1.23 $\times$ 10^–5^ cm^2^/V s for the MXene-assisted ETL ^34^. This value shows an order of magnitude increase compared to the bare SnO_2_ ETL. In the simulations, all parameters except for the MXene concentration in the ETL of the cells are kept constant. The values of carrier capture cross-section layers in PSCs with SnO_2_ ETL and MXene-added ETL are considered to be 1 $\times$ 10^–15^ cm^2^.

**Table 1:** Parameters used in the simulation by SCAPS for ITO/SnO_2_/CH_3_NH_3_PbI_3_/Spiro-OMeTAD/Ag PSCs structures.

Parameter	ITO	SnO_2_ (ETL)	CH_3_NH_3_PbI_3_ (absorber)	Spiro-OMeTAD (HTL)
$Thickness \, \left({\text{nm}}\right)/\varepsilon$	150^a^/8.9^b^	30^a^/9.0^b^	430^a^/10.0^e^	140^a^/3.0^f^
$E_g$ $/\chi \text{ (eV)}$	3.5^c^/4.5^c^	3.44^a^/4.39^a^	1.5^a^/3.93^a^	2.7^a^/2.5^a^
${N}_{c}/{N}_{v} (\times {10}^{19})\text{ (}{\text{cm}}^{-{3}}\text{)}$	0.22^c^/1.8^c^	0.22^b^/1.8^b^	0.1^e^/0.1^e^	10.0^f^/10.0^f^
${N}_{D}/{N}_{A} (\times {10}^{19})\text{ (}{\text{cm}}^{-{3}}\text{)}$	100/$-$	1.0/$-$	$-$/4$.0$	$-$/7.0
${N}_{t}(\times {10}^{15})\text{ (}{\text{cm}}^{-{3}}\text{)}$	1.0	1.0	0.001	0.1
${\mu }_{n}/{\mu }_{p} ({\text{cm}}^{2}/\text{V s})$	10.0^b^/10.0^b^	7.56 $\times {10}^{-6}$ ^a^/0.1^d^	2.0^f^/2.0^f^	2.0^f^/0.01^f^
*α* $\text{ (}{\text{cm}}^{-{1}}\text{)}$	From spectrum^i^			

^a^Ref. ^34^, ^b^Ref. ^46^, ^c^Ref. ^47^, ^d^Ref. ^48^, ^e^Ref. ^49^, ^f^Ref. ^50^, ^i^Ref. ^51,52^.

**Table 2:** Interfacial parameters used in the simulation by SCAPS for ITO/SnO_2_/CH_3_NH_3_PbI_3_/Spiro-OMeTAD/Ag PSCs structures.

Parameter	ITO/ETL	ETL/Absorber	Absorber/HTL
Defect type	Neutral	Neutral	Neutral
Capture cross section electron (cm^2^)	1.0 $\times {10}^{-19}$	1.0 $\times {10}^{-19}$	1.0 $\times {10}^{-19}$
Capture cross section hole (cm^2^)	1.0 $\times {10}^{-19}$	1.0 $\times {10}^{-19}$	1.0 $\times {10}^{-19}$
Energetic distribution	Single	Single	Single
Reference for defect energy level E_t_	Above the highest E_V_	Above the highest E_V_	Above the highest E_V_
Energy with respect to reference (eV)	0.6	0.6	0.6
Total density (cm^-2^)	1.0 $\times {10}^{12}$	1.0 $\times {10}^{9}$	1.0 $\times {10}^{9}$

## Results and discussion

**Figure 2:**
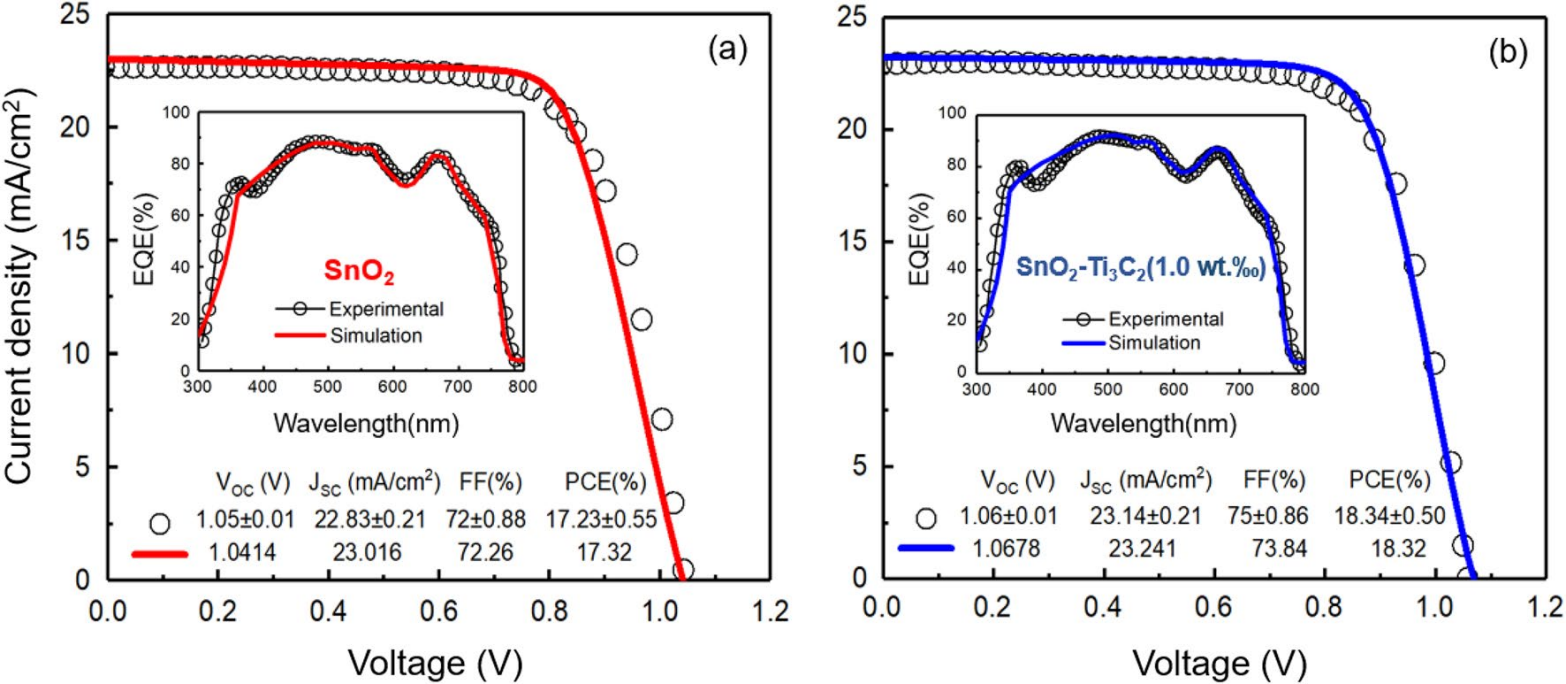
Simulated (solid line) and experimental (dotted) data of J–V and EQE curves of solar cells with different electron transport layers. (**a**) SnO_2_, (**b**) SnO_2_–Ti_3_C_2_ (1.0 wt‰).

The Fig. 2 illustrates the agreement between the experimental current density–Voltage (J–V) data for PSCs with SnO_2_ and SnO_2_-MXene (1.0 wt‰) ETLs and simulation results. In the same figure, the theoretical External Quantum Efficiency (EQE) curve closely matches the measured one. This indicates that the model was able to successfully explain the process of photovoltaics. It’s worth noting that the simulated photovoltaic parameters closely follow the measured values, as shown in the graph's inset.

**Figure 3:**
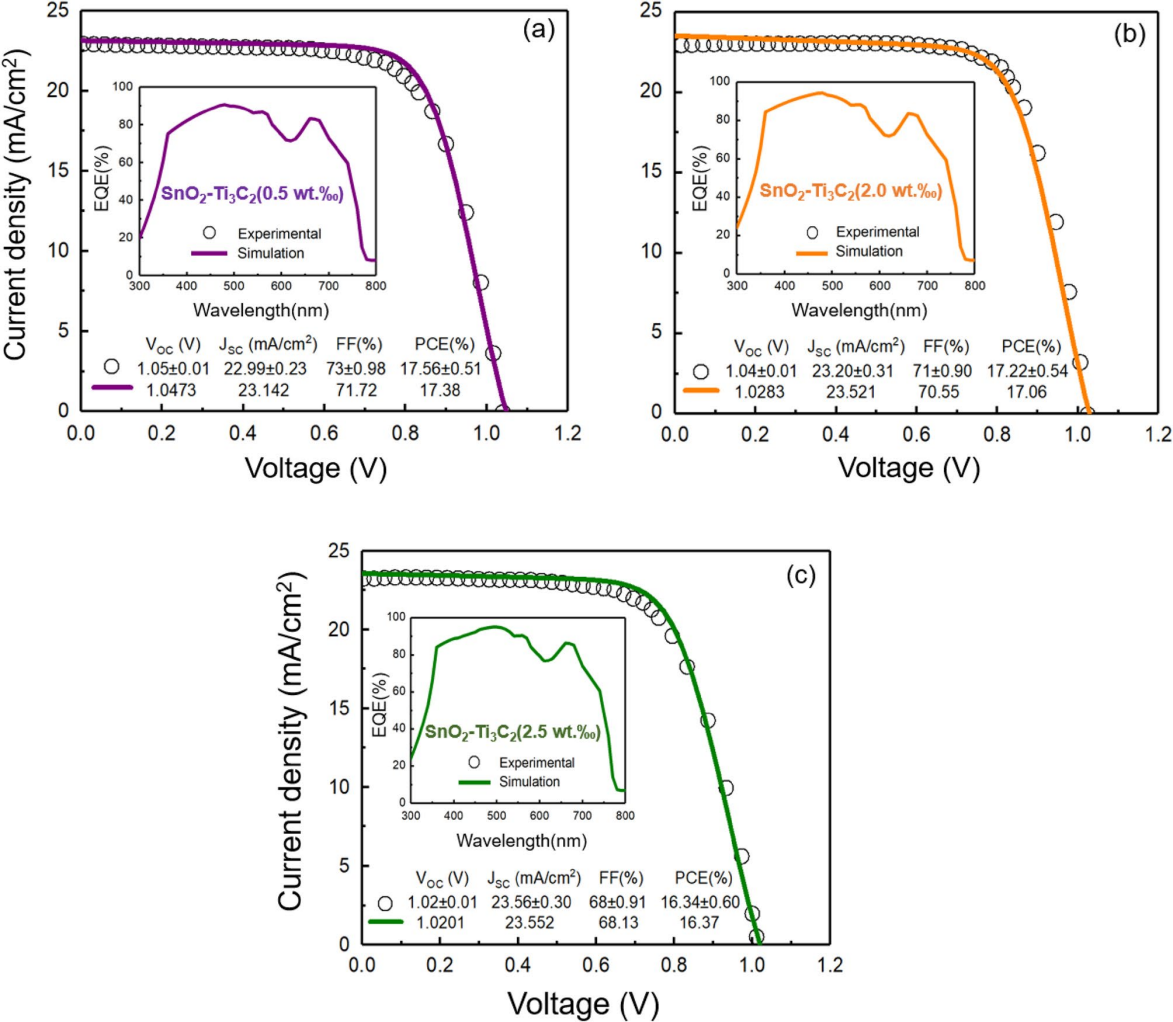
Simulated (solid line) and experimental (dotted) data of J–V and EQE curves of solar cells with different electron transport layers. (**a**) SnO_2_–Ti_3_C_2_ (0.5 wt‰), (**b**) SnO_2_-Ti_3_C_2_ (2.0 wt‰), and (**c**) SnO_2_–Ti_3_C_2_ (2.5 wt‰).

The original paper ^34^ does not provide EQE data for the PSCs of SnO_2_-MXene at varying concentrations (0.5, 1.5, 2.0, and 2.5 wt‰). We present simulated J-V curves for the samples, which closely match the experimental ones shown in Fig. 3. In this figure, the calculated photovoltaic characteristics obtained from the fitting are being compared with the measured ones, where a high degree of concurrency can be seen between them. The generated EQE curves are shown in the insets.

**Figure 4:**
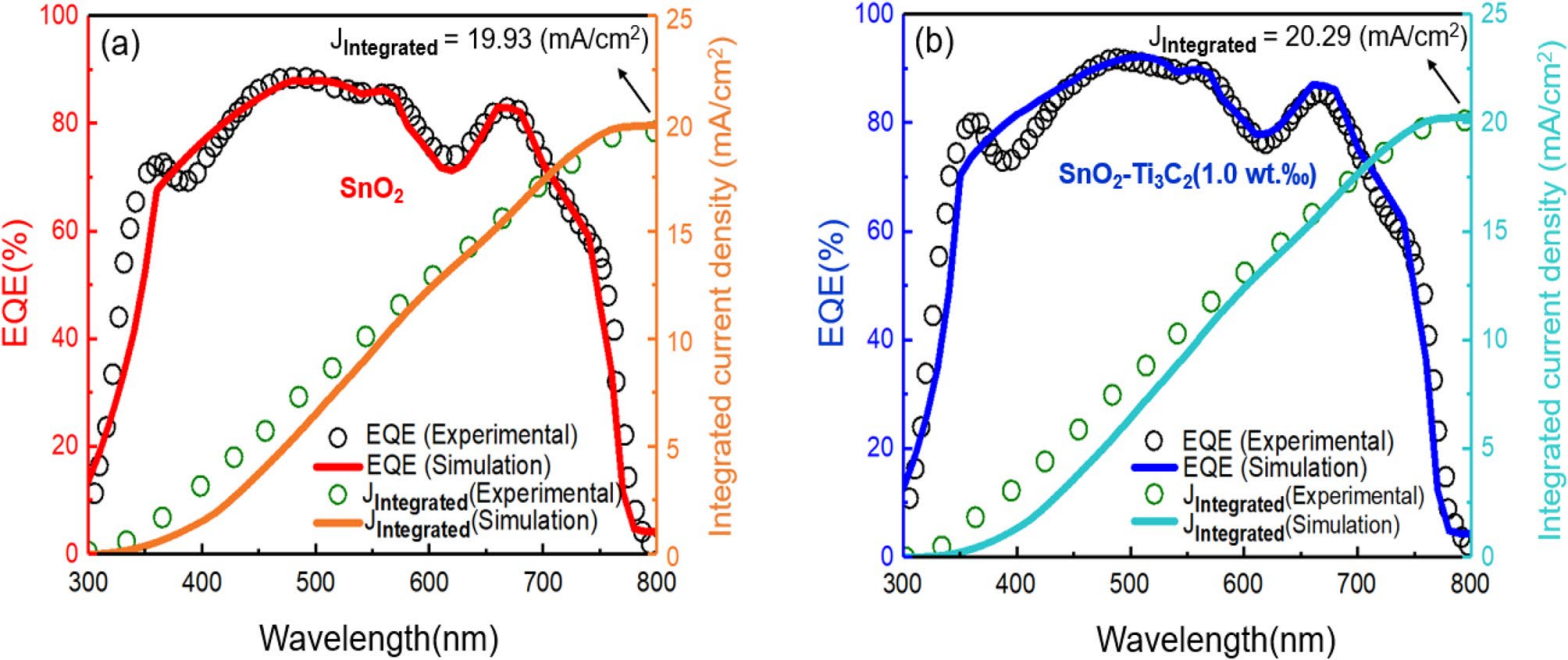
Simulated (solid line) and experimental (dotted) data of EQE spectra and the corresponding integrated current densities for the PSCs. (**a**) SnO_2_, (**b**) SnO_2_-Ti_3_C_2_ (1.0 wt‰).

**Figure 5:**
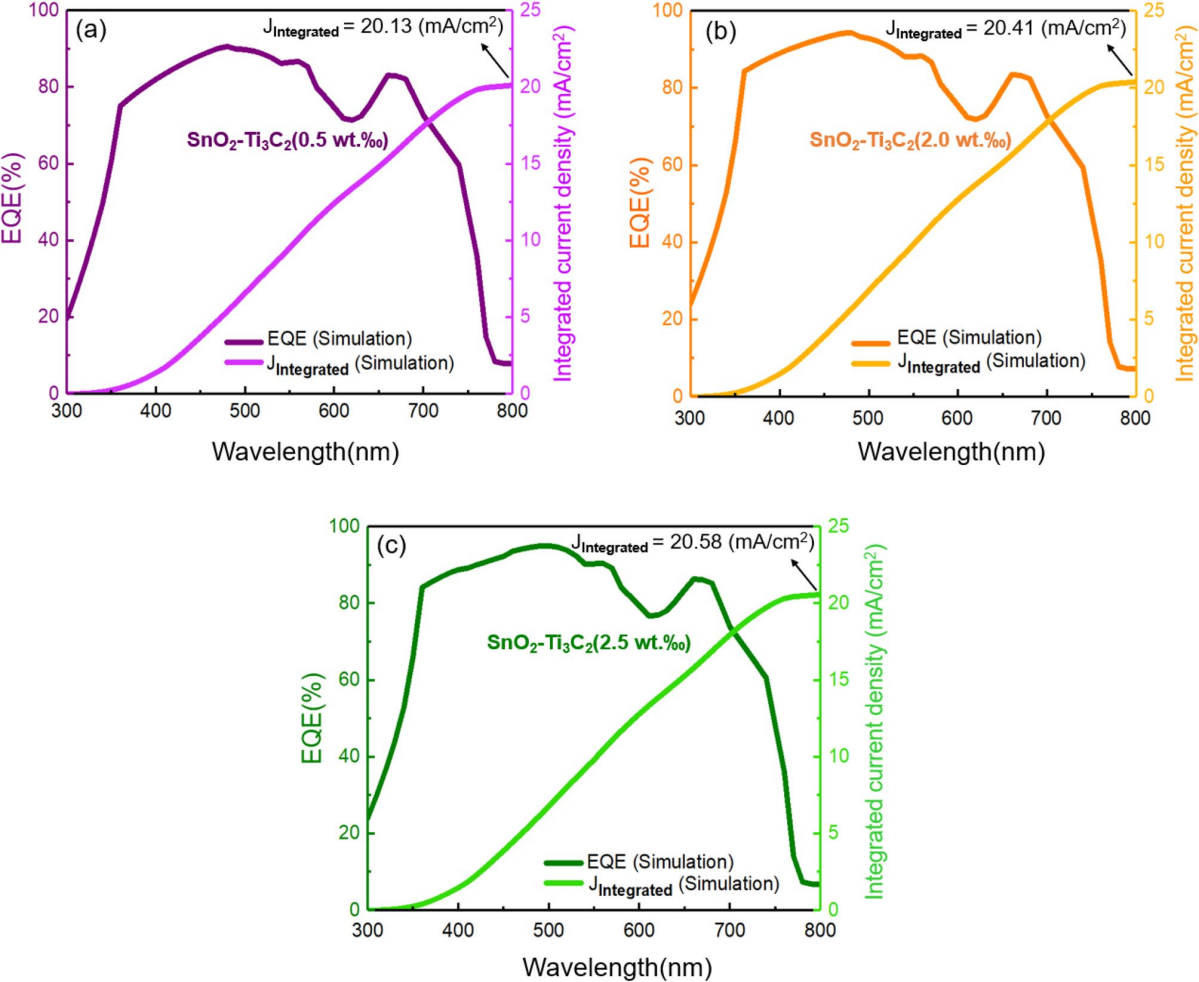
Simulated (solid line) data of EQE spectra and the corresponding integrated current densities for the PSCs. (**a**) SnO_2_–Ti_3_C_2_ (0.5 wt‰), (b) SnO_2_–Ti_3_C_2_ (2.0 wt‰), and (**c**) SnO_2_–Ti_3_C_2_ (2.5 wt‰).

For determining integrated current density, we combine the photon flow at a certain wavelength, leading to the flow of electrons leaving the solar cell at this wavelength ^53^. We have,

5 $${J}_{SC, EQE }= \text{ } -q{\int }_{{\lambda }_{0}}^{{\lambda }_{max}}EQE\left(\lambda \right){\Phi }_{ph, \lambda }d\lambda ,$$

where ${\Phi }_{ph, \lambda }$ is the photon flux of AM1.5. The simulated and experimental EQE spectra and their corresponding integrated currents density are depicted in Fig. 4. The calculated integrated current density for the SnO_2_-based cell is 19.93 mA cm^-2^. When 1.0 wt‰ MXene is added to the device, it increases to 20.29 mA cm^-2^. The deviation between the integrated current from EQE and the values obtained from the simulation of J_SC_ values (presented in Fig. 2) is around 10%. This indicates good accuracy of our J–V measured values. The integrated current density of SnO_2_–Ti_3_C_2_ (0.5 wt‰), SnO_2_–Ti_3_C_2_ (2.0 wt‰), and SnO_2_–Ti_3_C_2_ (2.5 wt‰) are 20.13 mA cm^-2^, 20.41 mA cm^-2^, and 20.58 mA cm^−2^, respectively. Figure 5 shows the result of the simulation of EQE and integrated current density for SnO_2_–Ti_3_C_2_ (0.5 wt‰), SnO_2_-Ti_3_C_2_ (2.0 wt‰), and SnO_2_–Ti_3_C_2_ (2.5 wt‰). It can be seen that the amount integrated current density follows the order of MXene weight percentage in the SnO_2_ ETL.

**Figure 6:**
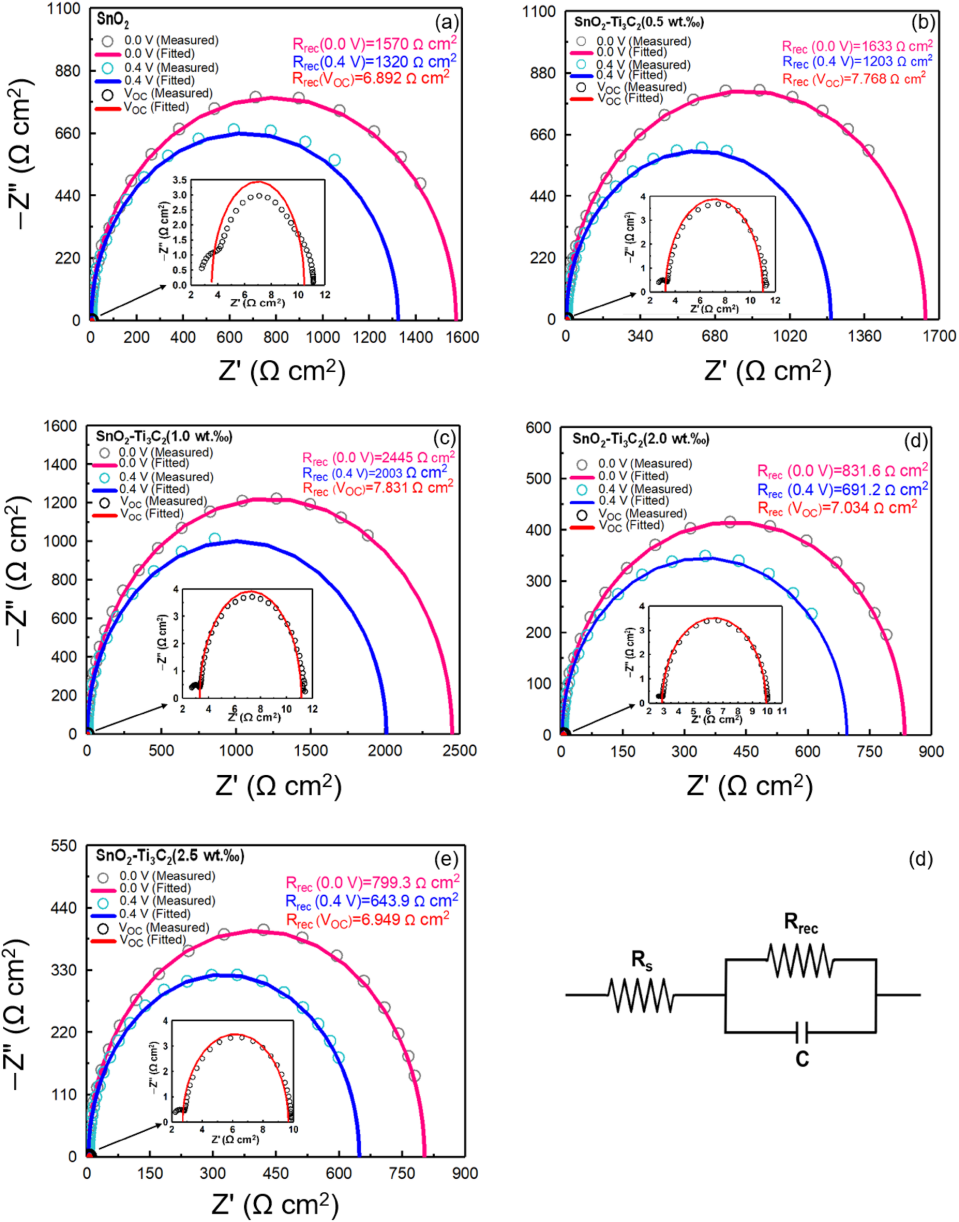
Measured (dotted) and fitted (solid line) data of Nyquist plots of PSCs fabricated with the different ETLs. (**a**) SnO_2_, (b) SnO_2_–Ti_3_C_2_ (0.5 wt‰), (**c**) SnO_2_–Ti_3_C_2_ (1.0 wt‰), (**d**) SnO_2_–Ti_3_C_2_ (2.0 wt‰), (**e**) SnO_2_–Ti_3_C_2_ (1.0 wt‰), and (**f**) Circuit representation of a fundamental relaxation process with characteristic resistance and capacitance.

The Nyquist plots of solar cells of different ETLs with recorded IS spectra are shown in Fig. 6. The ${R}_{rec}s$ calculated from fitting the semicircle Nyquist plots are shown in the same Fig. The semicircle is observed for all conditions, and it starts at a high frequency and ends at a low frequency. This semicircle can be fitted to an equivalent circuit. The wires and ITO substrate are largely associated with ${R}_{s}$. The main observed semicircle represents ${R}_{rec}$, and the interfacial capacitance (C) at the ETL/perovskite interface ^34^. ${R}_{s}$ is in series with other components and results in a shift in the Nyquist spectrum along the real axis away from the origin. The term ${R}_{rec}$ denotes the phenomenon of electron capture, where an electron or hole moves from the conduction or valence band to a defect in the bandgap or to surface states ^54,55^. Capacitance in IS corresponds to the storage of electrical energy. Physically, capacitance arises either due material polarisation (geometric capacitance), or due to local inhomogeneity in the distribution of free charge (electrochemical capacitance), usually related to charge dynamics. ${R}_{rec}$ is inversely proportional to charge recombination. Higher ${R}_{rec}$ suggests lower carrier recombination (better hole-blocking ability) ^34^. In Fig. 6, Nyquist plots are drawn for voltages of 0 V, 0.4 V and V_OC_. Among the PSCs of ETL with MXene, the resistance value of ${R}_{rec}$ is ordered as SnO_2_–Ti_3_C_2_ (1.0 wt‰) > SnO_2_–Ti_3_C_2_ (0.5 wt‰) > SnO_2_–Ti_3_C_2_ (2.0 wt‰) > SnO_2_–Ti_3_C_2_ (2.5 wt‰), where a higher resistance is better for electron collection. This implies that the least charge recombination occurs at the interface, resulting in the highest FF of SnO_2_–Ti_3_C_2_ (1.0 wt‰). This can be partly attributed to the better electron extraction due to the addition of Ti_3_C_2_. The performance improvement can practically be explained by ${R}_{rec}$. In general, higher ${R}_{rec}$ corresponds to higher PCE.

**Figure 7:**
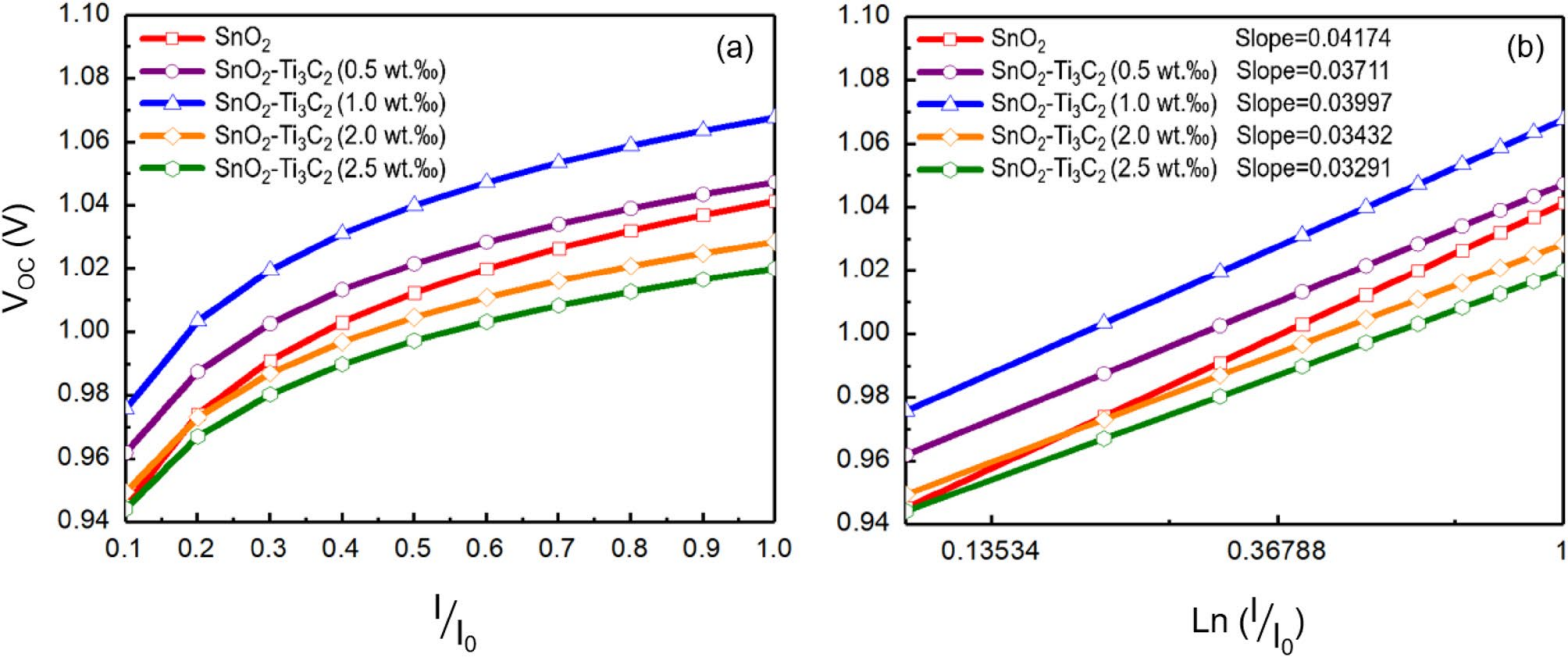
Plots of V_OC_ versus. (**a**) (I/I_0_), (**b**) the calculated slope of the V_OC_ versus ln (I/I_0_) curves for PSCs with SnO_2_ and MXene-assisted ETLs are shown.

Figure 7a displays the variation of V_OC_ values with illumination for cells with and without MXene. It is evident that the V_OC_ increases with the intensity of illumination; however, it almost reaches saturation at high light intensity. We used ^56,57^ to calculate the ${n}_{id}s$.

6 $${n}_{id}\text{ = }\frac{q}{kT}\frac{d{V}_{OC}}{dLn\left(\frac{I}{{I}_{0}} \right) } ,$$

where $k$ is Boltzmann’s constant, $T$ is temperature, $q$ is the elementary charge, $\frac{kT}{{q}}$ is the thermal voltage and is equal to $0.026 V$ at room temperature, and ${I}_{0}$ is reference intensity at one Sun.

**Figure 8:**
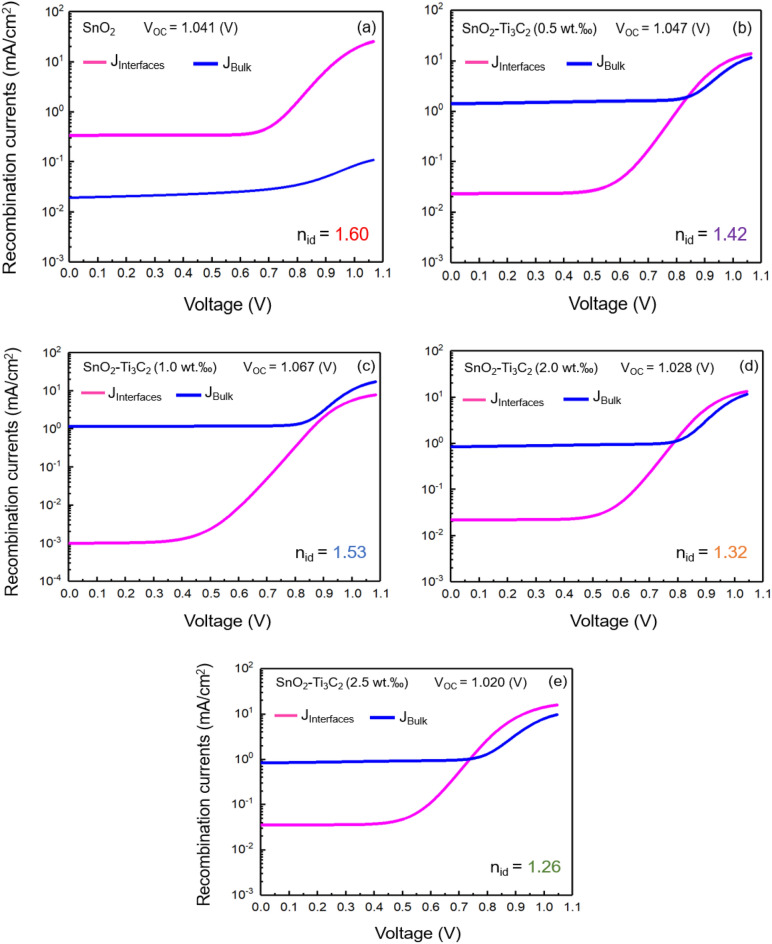
Bulk and interfacial recombination currents. The calculated open-circuit voltages and the ideality factors of each device are presented. (**a**) SnO_2_, (**b**) SnO_2_–Ti_3_C_2_ (0.5 wt‰), (**c**) SnO_2_–Ti_3_C_2_ (1.0 wt‰), (**d**) SnO_2_–Ti_3_C_2_ (2.0 wt‰), (**e**) SnO_2_–Ti_3_C_2_ (2.5 wt‰).

Figure 7b displays the curves of V_OC_ changes against Ln(I/I_0_) and the slopes obtained for calculating the n_id_ values. The bulk and interfacial Shockley–Read–Hall (SRH) recombination are formulated according to Refs.^58,59^.

7 $${{R}_{SRH}} \, = \text{ } {\sigma }_{n, p}\times {{N}_{t}}\, \times {\upsilon }_{th} \, \frac{np-{n}_{i}^{2} \, }{n+p+2{n}_{i} \, {\text{cos}}h\left( \frac{{E}_{t}-{E}_{i}}{kT}\right) \, } ,$$

8 $${\tau }_{n, p}\text{=}\frac{1}{{\sigma }_{n, p} \, \times {{N}_{t}}\times {\upsilon }_{th}} ,$$

where ${n}_{i}$ is the equilibrium charge density, ${\sigma }_{n, p}$ is the electron and hole absorption cross-section, and $n (p)$ is electron (hole) density under the non-equivalence condition. ${E}_{i}$ and ${E}_{t}$ represent the intrinsic and trap defect energy levels, respectively, ${\upsilon }_{th}$ represents the thermal velocity and ${\tau }_{n, p}$ is the carrier lifetime. In Fig. 8, we can see the bulk and interfacial recombination currents, as well as cap ${V}_{OC}s$, and calculated ${n}_{id}s$. It’s worth noting that band-to-band recombination was found to be negligible. In the PSC with bare ETL of MXene, the ideality factor is relatively close to 2 $({n}_{id}=1.60)$.

When additive MXenes are introduced into the ETL of solar cells, the ideality factor values become closer to 1. This indicates that the interfacial mechanism, rather than bulk recombination, dominates in the MXene-assisted ETL cells. Among these cells, the one with 1.0 wt‰ of MXene shows the highest ideality factor and V_OC_ values. This suggests that adding 1.0 wt‰ of MXene into the SnO_2_ ETL makes the interface recombination least effective, resulting in the best cell performance.

**Figure 9:**
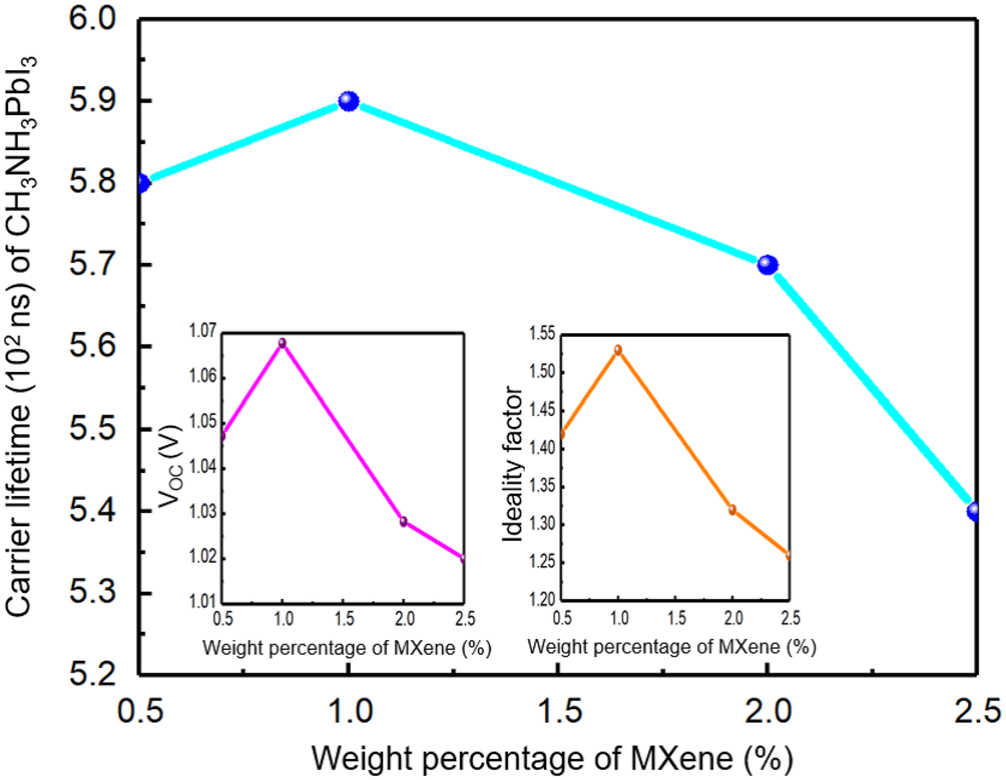
Carrier Lifetime of absorber layer curve vs MXene weight percentage (0.5, 1.0, 2.0, 2.5 wt‰). Two insets depict the calculated V_OC_ and ideality factors varying with MXene weight percentage used in ETL.

**Figure 10:**
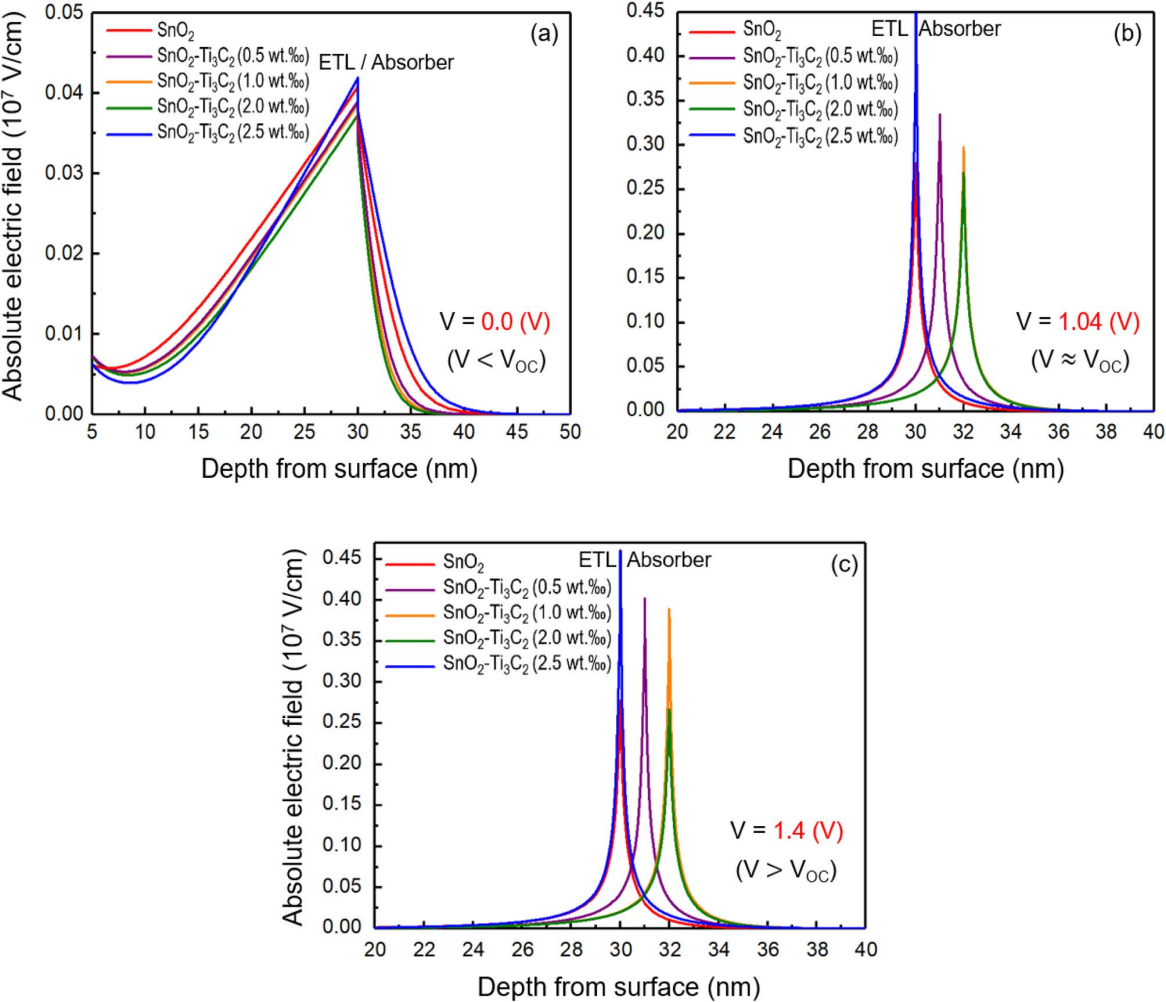
The Absolute electric field at the ETL/Absorber interface for V < V_OC_ (**a**), V ≈ V_OC_ (**b**), and V > V_OC_ (**c**).

It is well established that incorporating MXenes in the structure of PSCs can enhance their performance. However, it is important to note that increasing the weight percentage of MXene in the SnO_2_ ETL beyond 1.0 wt‰ may lead to a reduction in the V_OC_, which requires further discussion. This same observation also applies to cells with 0.5 wt‰ of MXene. To remove ambiguities, we plotted the carrier lifetime of the absorber layer against the weight percentage of MXene in ETL (Fig. 9). It can be observed that the carrier lifetime reaches its peak when 1.0 wt‰ MXene is present in the SnO_2_ ETL structure. This indicates that the charge carriers generated in this cell will have a longer effective time for extraction by the charge transport layers, in comparison to the other cells. In Fig. 10, the electric field distribution of cells at the ETL/absorber interface was studied for applied voltages that were less than, equal to, and greater than the V_OC_. It was observed that the 1.0 wt‰ MXene-added cell had a stronger electric dipole formed at the ETL/absorber interface, which could establish a significant potential difference across the ETL. This would lead to a shorter extraction time for the electrons from the ETL, as compared to the charge carrier lifetime in the absorber layer ^34,60,61^. As a result, the photogenerated charge carriers in the absorber layer could be extracted immediately. On the other hand, due to the weaker dipole moment of the other cells formed at the ETL/absorber, the photogenerated carriers took much more time to be collected. It has been observed that the addition of MXene to SnO_2_ ETL cells results in higher recombination, which significantly reduces their overall performance. However, it has been found that the MXene-assisted cells with 1.0 wt‰ MXene-added-SnO_2_ ETL show better performance compared to other such cells. This is due to the fact that interfacial recombination plays a more crucial role than bulk recombination in determining cell performance, as indicated by the higher ideality factor of these cells. In cells containing MXene, there is a correlation between n_id_s and cell performance. This correlation can be explained through the inset in Fig. 9, where the curves of V_OC_ and n_id_ versus the MXene weight percentage follow the same pattern as the carrier lifetime in the absorber layer. Therefore, in these cells, a lower n_id_ indicates a higher interfacial recombination current, resulting in a less efficient cell.

**Figure 11:**
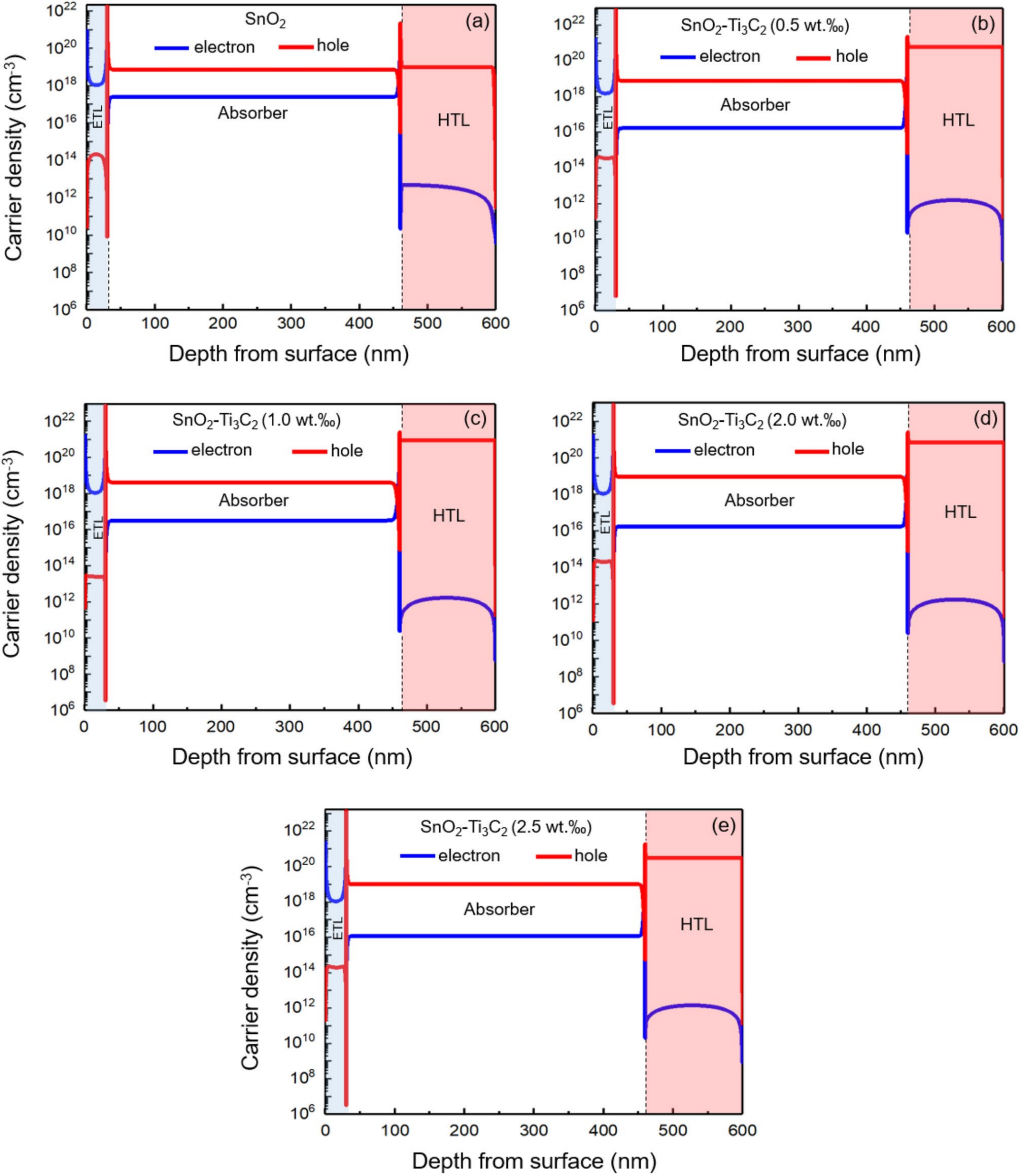
Charge carrier density across the absorber and charge transport layers of the cells with and without MXene. (**a**) SnO_2_, (b) SnO_2_–Ti_3_C_2_ (0.5 wt‰), (**c**) SnO_2_–Ti_3_C_2_ (1.0 wt‰), (**d**) SnO_2_–Ti_3_C_2_ (2.0 wt‰), (**e**) SnO_2_–Ti_3_C_2_ (2.5 wt‰).

In literature, the correlation between quasi-Fermi level splitting (QFLS) and charge carrier densities (n and p) in the absorber layer has been discussed (62).

9 $$QFLS=k_{{B}}T{Ln}\left(\frac{np}{{n}_{i}^{2}}\right)\text{ = }k_{{B}}T {{Ln}}\left(\frac{n\beta }{{n}_{i}^{2}}\right)+const,$$

where *β* is a parameter defining the relationship between the carrier density and the perturbation of the QFLs from equilibrium and is equal to 1 or 2, and ${n}_{i}$ is the equilibrium charge density. In brief, if the charge carrier densities undergo the condition $n$ ≈ $p$, the bulk recombination will dominate. Meanwhile, the presence of a dominant charge carrier, e.g., $n$ >> $p$ (or $p$ >> $n$), makes the interfacial recombination the dominant mechanism. Figure 11 shows $n$ and $p$ for cells with and without MXene, and Table 3 provides values of these values at the middle of the absorber layer. In the cell with bare SnO_2_ ETL shows better $n$ ≈ $p$ is condition compared to the other cells. This indicated that bulk recombination dominates and ${n}_{id}$ is likely to be close to 2. On the other hand, in MXene-assisted cells, the condition $n$ >> $p$ is satisfied, and the ${n}_{id}$ is relatively close to 1. Among these cells, the one with 1.0 wt‰ of MXene has the lowest $p$/$n$ ratio, which confirms the highest ${n}_{id}$.

**Table 3:** Electron (hole) density values in the PSCs.

ETLs	P	n	p/n
SnO_2_	7.2 $\times$ 10^18^	2.4 $\times$ ${10}^{17}$	3.0 $\times {10}^{1}$
SnO_2_–Ti_3_C_2_ (0.5 wt‰)	7.6 $\times {10}^{18}$	1.7 $\times {10}^{16}$	4.5 $\times {10}^{2}$
SnO_2_–Ti_3_C_2_ (1.0 wt‰)	4.0 $\times$ ${10}^{18}$	3.1 $\times {10}^{16}$	1.3 $\times {10}^{2}$
SnO_2_–Ti_3_C_2_ (2.0 wt‰)	9.0 $\times {10}^{18}$	1.7 $\times$ ${10}^{16}$	5.3 $\times {10}^{2}$
SnO_2_–Ti_3_C_2_ (2.5 wt‰)	1.0 $\times {10}^{19}$	1.2 $\times {10}^{16}$	8.3 $\times {10}^{2}$

After gaining more insights into the role of incorporated MXenes into the SnO_2_ ETL, we assessed the photocurrent density using Eq. (10), where $J\left(V\right)$ and ${J}_{dark}$ are the current density under light and the dark current density, respectively ^62,63^.

10 $${J}_{ph}=J\left(V\right)-{J}_{dark }.$$

The electric field established in the absorber layer is $E=\frac{{V}_{bi}-V}{d}$, where ${V}_{bi}$ is the built-in potential, and $d$ is the thickness ^61^. The drift caused by such an internal electric field makes a photogenerated current. This photogenerated current ${J}_{ph} (V)$ is formulated as ^64,65^,

$${J}_{ph}\left(V\right)=\left|{J}_{sc}\right| if \mu \tau \frac{{V}_{bi}-V}{d} >d,$$

$${J}_{ph}\left(V\right)=-\left|{J}_{sc}\right| if \mu \tau \frac{V-{V}_{bi}}{d} >d,$$

11 $${J}_{ph}\left(V\right)=\left|{J}_{sc}\right|\frac{\mu \tau \left({V}_{bi}-V\right)}{{d}^{2}} else,$$

where $\mu$ is charge carrier mobility, *τ* is charge carrier lifetime, *V* is the applied voltage, and $d$ is the sample thickness. The equation above provides a practical method to determine the built-in potential by finding the intersection of the ${J}_{ph}\left(V\right)$ curve with the voltage axis.

**Figure 12:**
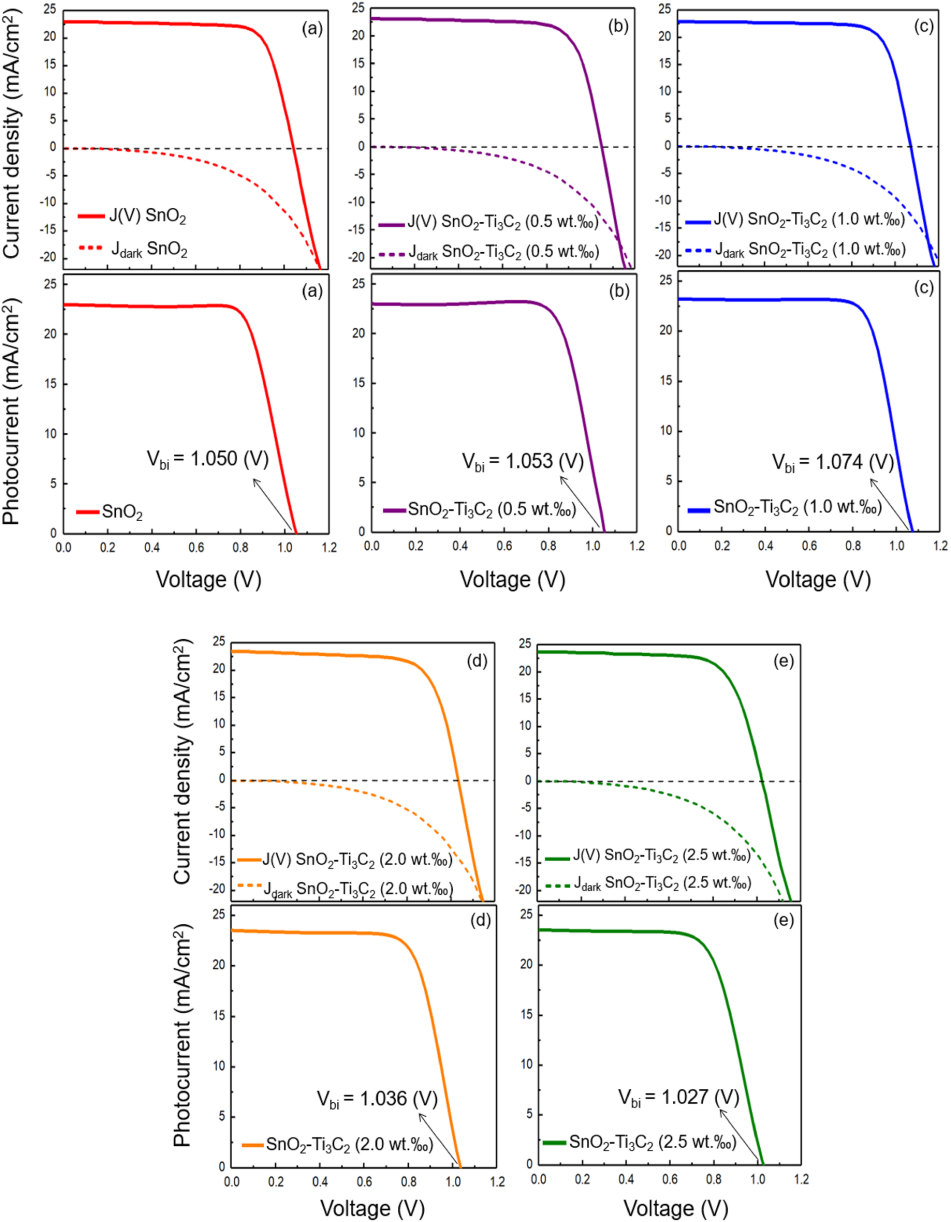
The current and photocurrent densities of PSCs with different ETLs. (**a**) SnO_2_, (**b**) SnO_2_–Ti_3_C_2_ (0.5 wt‰), (**c**) SnO_2_–Ti_3_C_2_ (1.0 wt‰), (**d**) SnO_2_–Ti_3_C_2_ (2.0 wt‰), (**e**) SnO_2_–Ti_3_C_2_ (2.5 wt‰).

Figure 12 illustrates the ${J}_{ph}\left(V\right)$ of the cells with and without MXene in their ETL structure. This figure clarifies how to derive the ${V}_{bi}$. for each sample. Improving the ${V}_{OC}$ is crucial in photovoltaic structures as it effectively reduces interfacial recombination. On the other hand, the voltage limit of the ${V}_{OC}$ is determined by the ${V}_{bi}$ which is vital for achieving better cell performance ^66–70^. As depicted in the figure, the sample containing 1.0 wt.‰ MXene in the ETL displays the highest ${V}_{bi}$, which is why it has the highest PCE among all samples.

In the last part of this work, an optimization procedure of the SnO_2_–Ti_3_C_2_ (1.0 wt‰) is presented, and it is hoped that the results of this optimization will have a significant impact on practical features of photovoltaic science, as well as understanding the role of the thickness of the layers.

**Figure 13:**
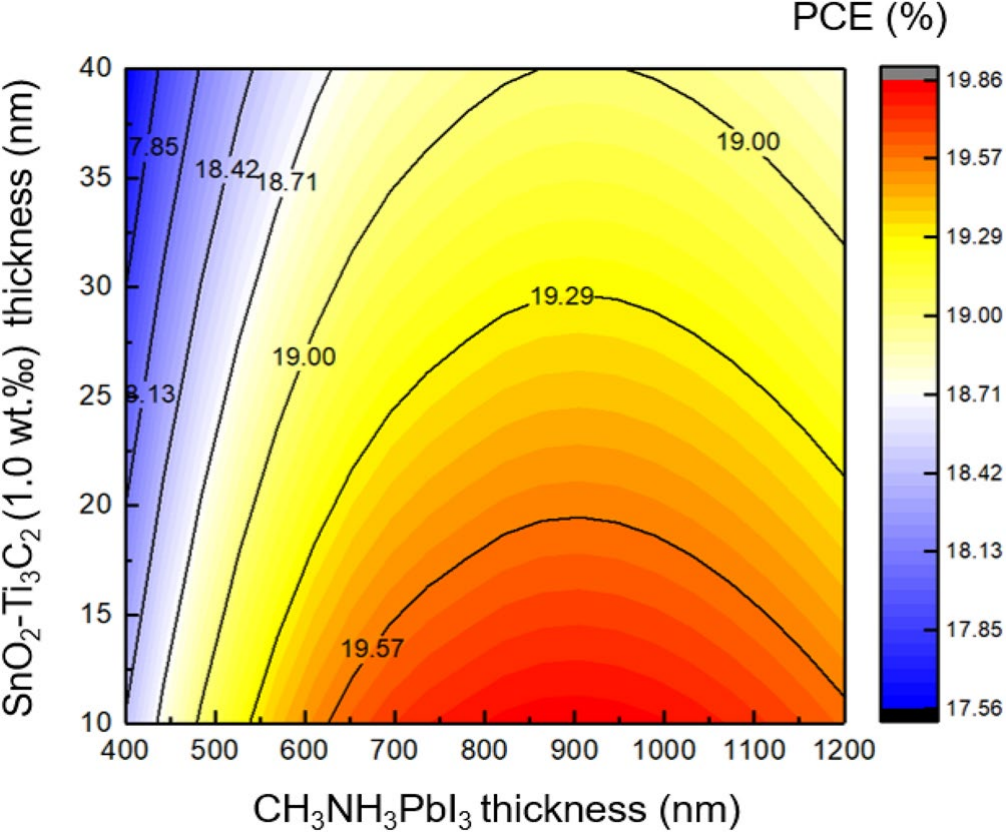
The effect of the SnO_2_–Ti_3_C_2_ (1.0 wt‰) ETL and absorber thickness variation on the cell performance.

Figure 13 shows a contour plot that displays the variation of the thickness of the SnO_2_–Ti_3_C_2_ (1.0 wt‰) ETL and the absorber layer, ranging from 10 to 40 nm and 400 nm to 1200 nm, respectively. The optimal thickness for the absorber layer is 700 nm, while the optimal thickness for the ETL is 10 nm. We will use these values for the ETL and absorber thickness parameters throughout the optimization process.

**Figure 14:**
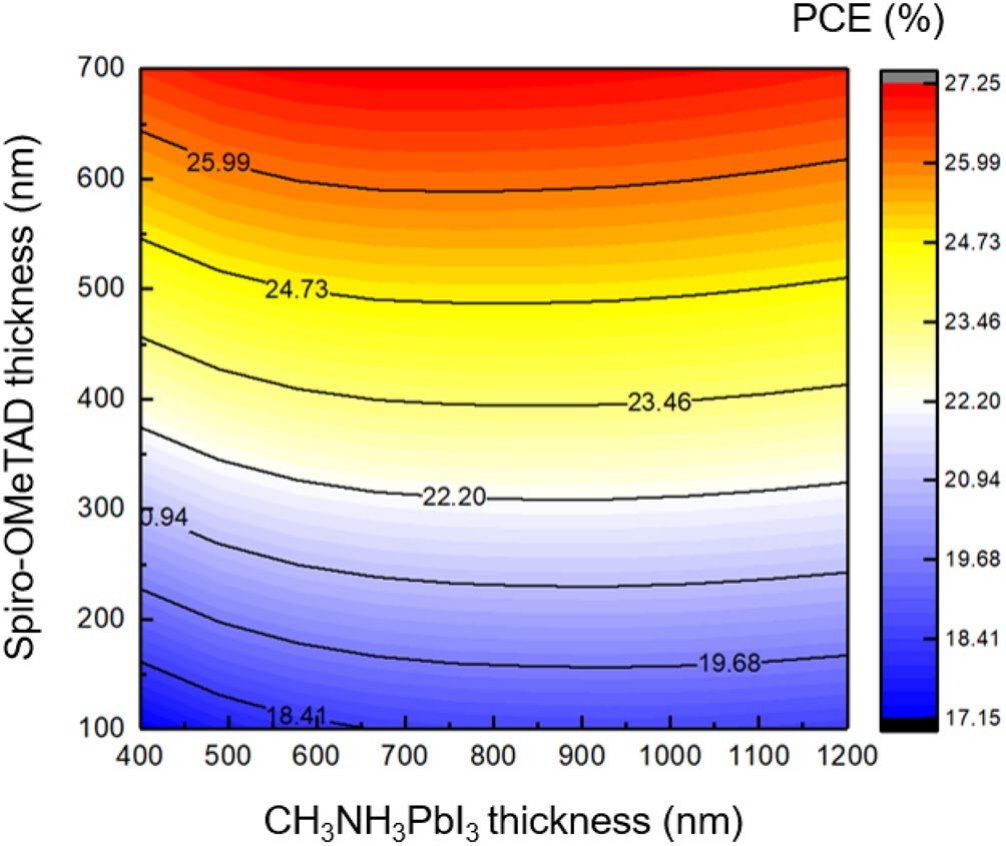
The effect of the HTL and absorber thickness variation on the cell performance.

Figure 14 illustrates the relationship between the thickness of the HTL and the absorber. The range of HTL layer thickness considered in this study is between 100 and 700 nm. After optimization, a thickness of 700 nm was selected as the most suitable for the HTL layer thickness.

**Figure 15:**
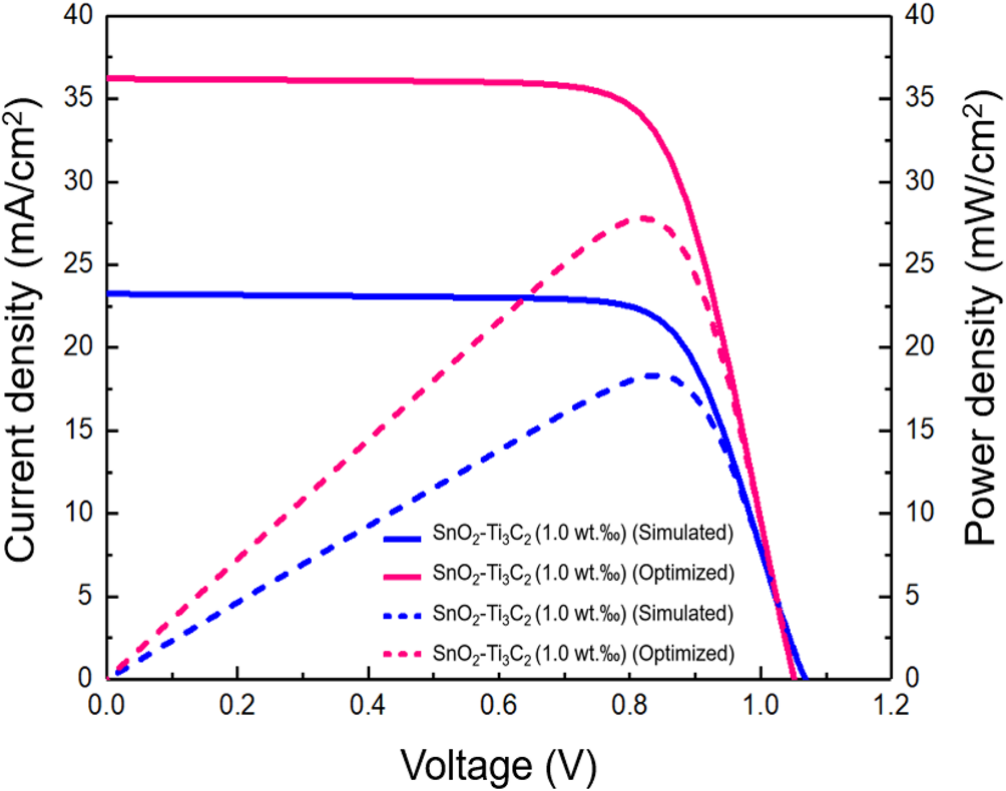
J–V (solid lines) and P–V (dashed lines) for the MXene-assisted cell with SnO_2_–Ti_3_C_2_ (1.0 wt‰) ETL.

Figure 15 displays the J–V and power density–voltage (P–V) curves, which were plotted based on the optimized parameters discussed earlier for the MXene-assisted cell with SnO_2_–Ti_3_C_2_ (1.0 wt‰) ETL. The photovoltaic response of the optimized MXene-assisted cell with SnO_2_–Ti_3_C_2_ (1.0 wt‰) ETL was achieved through J_SC_ = 36.21 mA/cm^2^, V_OC_ = 1.051 (V), FF = 73.07% and PCE = 27.81%, respectively.

## Conclusion

A numerical analysis was conducted on devices with and without 2D MXene in their SnO_2_ ETLs using SCAPS-1D software. The study found that a device architecture of ITO/ETL/CH_3_NH_3_PbI_3_/Spiro-OMeTAD/Ag with SnO_2_–Ti_3_C_2_ (1.0 wt‰) as the ETL achieved a relatively high PCE of 27.81%. It is believed that the added MXene plays a crucial role in reducing the interfacial recombination, which is the primary reason for the improved performance of the cell. By calculating the ideality factor (n_id_), we established a correlation between this quantity and the cell performance. We found that the sample with the highest efficiency also had the highest n_id_ value of 1.53. The improvement in efficiency in PSCs can be credited to the increase in R_rec_, a parameter that explains the enhancement in efficiency. This parameter demonstrates that IS is an easy and alternative technique for obtaining information about PSCs.

## Data Availability

The datasets generated during and/or analysed during the current study are available from the corresponding author on reasonable request.
